# Identifying Good Candidates for Active Surveillance of Ductal Carcinoma *In Situ*: Insights from a Large Neoadjuvant Endocrine Therapy Cohort

**DOI:** 10.1158/2767-9764.CRC-22-0263

**Published:** 2022-12-07

**Authors:** Alexa C. Glencer, Phoebe N. Miller, Heather Greenwood, Cristian K. Maldonado Rodas, Rita Freimanis, Amrita Basu, Rita A. Mukhtar, Case Brabham, Paul Kim, E. Shelley Hwang, Jennifer M. Rosenbluth, Gillian L. Hirst, Michael J. Campbell, Alexander D. Borowsky, Laura J. Esserman

**Affiliations:** 1 Department of Surgery, University of California San Francisco, San Francisco, California.; 2 University of California San Francisco School of Medicine, San Francisco, California.; 3 Department of Radiology, University of California San Francisco, San Francisco, California.; 4 Harvard Medical School, Boston, Massachusetts.; 5 Quinnipiac University School of Medicine, North Haven, Connecticut.; 6 Department of Surgery, Duke University, Durham, North Carolina.; 7 Department of Medicine, University of California San Francisco, San Francisco, California.; 8 Department of Pathology, University of California Davis, Sacramento, California.

## Abstract

Ductal carcinoma *in situ* (DCIS) is a biologically heterogenous entity with uncertain risk for invasive ductal carcinoma (IDC) development. Standard treatment is surgical resection often followed by radiation. New approaches are needed to reduce overtreatment. This was an observational study that enrolled patients with DCIS who chose not to pursue surgical resection from 2002 to 2019 at a single academic medical center. All patients underwent breast MRI exams at 3- to 6-month intervals. Patients with hormone receptor–positive disease received endocrine therapy. Surgical resection was strongly recommended if clinical or radiographic evidence of disease progression developed. A recursive partitioning (R-PART) algorithm incorporating breast MRI features and endocrine responsiveness was used retrospectively to stratify risk of IDC. A total of 71 patients were enrolled, 2 with bilateral DCIS (73 lesions). A total of 34 (46.6%) were premenopausal, 68 (93.2%) were hormone-receptor positive, and 60 (82.1%) were intermediate- or high-grade lesions. Mean follow-up time was 8.5 years. Over half (52.1%) remained on active surveillance without evidence of IDC with mean duration of 7.4 years. Twenty patients developed IDC, of which 6 were HER2 positive. DCIS and subsequent IDC had highly concordant tumor biology. Risk of IDC was characterized by MRI features after 6 months of endocrine therapy exposure; low-, intermediate-, and high-risk groups were identified with respective IDC rates of 8.7%, 20.0%, and 68.2%. Thus, active surveillance consisting of neoadjuvant endocrine therapy and serial breast MRI may be an effective tool to risk-stratify patients with DCIS and optimally select medical or surgical management.

**Significance:**

A retrospective analysis of 71 patients with DCIS who did not undergo upfront surgery demonstrated that breast MRI features after short-term exposure to endocrine therapy identify those at high (68.2%), intermediate (20.0%), and low risk (8.7%) of IDC. With 7.4 years mean follow-up, 52.1% of patients remain on active surveillance. A period of active surveillance offers the opportunity to risk-stratify DCIS lesions and guide decisions for operative management.

## Introduction

Ductal carcinoma *in situ* (DCIS) is a potentially preinvasive neoplasm associated with an increased risk for the development of both ipsilateral and contralateral invasive breast cancer. The widespread implementation of breast cancer screening has increased incidence of these lesions from 3% to 25% of all breast cancers detected (1). However, the removal of 50,000 to 60,000 DCIS lesions annually has not been accompanied by a reduction in the incidence of invasive breast cancers, leading to concern that many DCIS lesions are overtreated (1–3). Thus, it is critical that we improve our understanding of the natural history of DCIS and refine management by identifying novel methods to differentiate those most likely to develop or harbor invasive disease who are good candidates for surgery from those with reversible or indolent biology who do not benefit from surgery (1, 4).

The risk for development of invasive ductal carcinoma (IDC) in the absence of therapy is estimated to be 14%–45% (5, 6). Standard treatment for DCIS is either mastectomy or lumpectomy and radiation; systemic hormonal therapy is offered to those with hormone receptor–positive (HR^+^) disease (7, 8). Following lumpectomy alone, the risk of recurrence is 15%–19% (9, 10). The combination of breast radiation and endocrine therapy can reduce the risk of recurrence in the first 5 years by greater than 50% (11–14). However, among patients with HR^+^ DCIS who have received radiation and endocrine therapy, the risk of contralateral events after 15 years is equal to the risk of ipsilateral events (9). This suggests that the presence of DCIS in some women could represent a high-risk environment rather than a focal risk amenable to surgical resection. Endocrine risk–reducing therapy may be an effective means of preventing breast cancer recurrence both ipsilaterally and contralaterally.

In MRI, background parenchymal enhancement (BPE) describes a phenomenon in which normal breast tissue shows signal enhancement related to uptake of intravenous contrast. Biologically, BPE is believed to represent tissue activated by endogenous hormones, primarily estrogen, and has been shown to be an independent marker of breast cancer risk (15). It may also serve as an imaging biomarker of treatment response to chemotherapy and hormonal agents. Thus, BPE has the potential to function as an intermediate endpoint for assessing effectiveness of interventions to reduce risk.

Fewer than 2% of patients diagnosed with DCIS elect to omit surgery (6). Here, we present a retrospective analysis of a large cohort of women diagnosed with DCIS at the University of California San Francisco (UCSF, San Francisco, CA) who declined surgery but consented to be followed over time with intensive surveillance. Using long-term outcomes of active surveillance, we evaluate whether MRI features identified early in the course of care, after short-term exposure to endocrine therapy, can be used to stratify risk for development of invasive breast cancer. This study has informed the development of parameters and inputs for a prospective multicenter active surveillance study.

## Materials and Methods

### Clinical Cohort

At UCSF (San Francisco, CA), we have maintained a database of patients enrolled in imaging studies for DCIS that includes women who were unwilling to undergo surgical resection at diagnosis and were subsequently assigned to a protocol of intensive serial imaging surveillance. Written informed consent was obtained from all patients enrolled in these studies to have their clinical, imaging, and pathologic data evaluated for research purposes in accordance with the Declaration of Helsinki and with approval of the UCSF (San Francisco, CA) Institutional Review Board. All patients underwent active surveillance consisting of examinations and imaging every 3 to 6 months, and all HR^+^ patients were offered endocrine therapy. In general, after 2 years, imaging was reduced from every 3 to every 6 months if a patient's clinical course was stable. Surgical resection was recommended for patients who had a persistent, enlarging, or new mass (a feature known to be associated with invasive rather than *in situ* disease; ref. 16). Most, but not all, patients followed their breast surgeon's recommendation to proceed with surgery when suspicion of disease progression developed.

### Study Design

In this retrospective analysis, we sought to determine whether features on serial MRI could be identified that select women who are likely to avoid IDC with active surveillance. We searched the UCSF DCIS imaging database, which includes patients on studies that required surgery after 3 or 6 months of therapy as well as patients who declined surgery at diagnosis and were consented to a serial imaging protocol. We included the latter patients if they had at least two breast MRIs and at least 2 years of follow-up from the time of diagnosis. Demographic data, MRI reports, operative reports, surgical pathology, recurrence events, and follow-up duration were obtained from patient medical records. Physician recommendations regarding the safety of continuing active surveillance versus strongly recommending surgical management were also abstracted from the medical record by a breast surgeon (R.A. Mukhtar). The primary outcome was the development or identification of invasive cancer. R-PART (see below) was retrospectively used to determine whether MRI features over the first 3 to 6 months of endocrine therapy could segregate groups of patients who were good candidates for continued active surveillance from those who were likely to develop invasive disease.

### Endocrine Risk Reduction

Endocrine risk–reduction therapy was recommended for patients with HR^+^ DCIS. Premenopausal women were offered tamoxifen (20 mg/day) and postmenopausal women offered a standard dose of an aromatase inhibitor with the recommendation to continue therapy for at least 5 years.

### Imaging Protocol

Serial breast MRIs were conducted at the time of initial DCIS diagnosis (baseline), at 1 month, 6 months, and then repeated every 3 to 6 months. Over time, the surveillance strategy changed to harmonize with CALGB 40903, a trial of neoadjuvant endocrine exposure in DCIS with MRIs performed at 0 and 3 months (17).

Breast MRIs were performed on 1.5T or 3.0T magnets at UCSF (San Francisco, CA). Each study included the standard breast MRI sequences of fat-suppressed T2W, nonfat-suppressed T1W, fat-suppressed precontrast T1W, and fat-suppressed T1W postcontrast images with at least two postcontrast timepoints.

Retrospectively, two breast radiologists (H. Greenwood, R. Freimanis) analyzed all breast MRIs for presence of a mass [vs. non-mass enhancement (NME)], lesion conspicuity, BPE, and change in BPE at each MRI timepoint. BPE was defined as minimal, mild, moderate, or marked, and endocrine responsive states were captured with serial MRIs. Both radiologists were blinded to clinical outcomes and other imaging.

### IHC and Molecular Profiling

Estrogen receptor (ER) and progesterone receptor (PR) results, performed using routine clinical protocols on core biopsy and surgical resection specimens, were collected from chart review. Standard HER2 IHC and FISH, if necessary, were performed on blocks collected from initial DCIS core biopsies and surgical specimens when they could be obtained. Invasive cancers that developed had standard receptor testing with ER, PR, HER2, and molecular profiling with Mammaprint (18) and Blueprint (Agendia; ref. 19).

### R-PART Analysis

R-PART is a form of decision tree analysis that can classify a population into homogenous subpopulations according to the association between a set of independent variables and a dependent variable. This R-PART tool finds groupings of the independent values that best predict a dependent variable value. These partitions are done recursively until a form of the tree with the desired fit is reached. The optimal partitions are chosen from all possible partition options (20).

R-PART was applied to this cohort of patients with DCIS to divide the cohort according to MRI features at baseline and after response to endocrine therapy that were most predictive of developing IDC. MRI features that were fed into the R-PART algorithm included BPE, change in BPE, how distinct the lesion is from BPE, likelihood of baseline invasive cancer, lesion change since prior MRI, likelihood of new or progressed DCIS, and likelihood of new or progressed invasive cancer. Performance of the model was assessed through root node error [percent of correctly sorted records at the first (root) splitting node] (error = 0.27) multiplied by the cross-validation error (average error = 1.0), a predictive measure of accuracy.

### Data Availability Statement

The data generated in this study are available upon request from the corresponding author.

## Results

**FIGURE 1:**
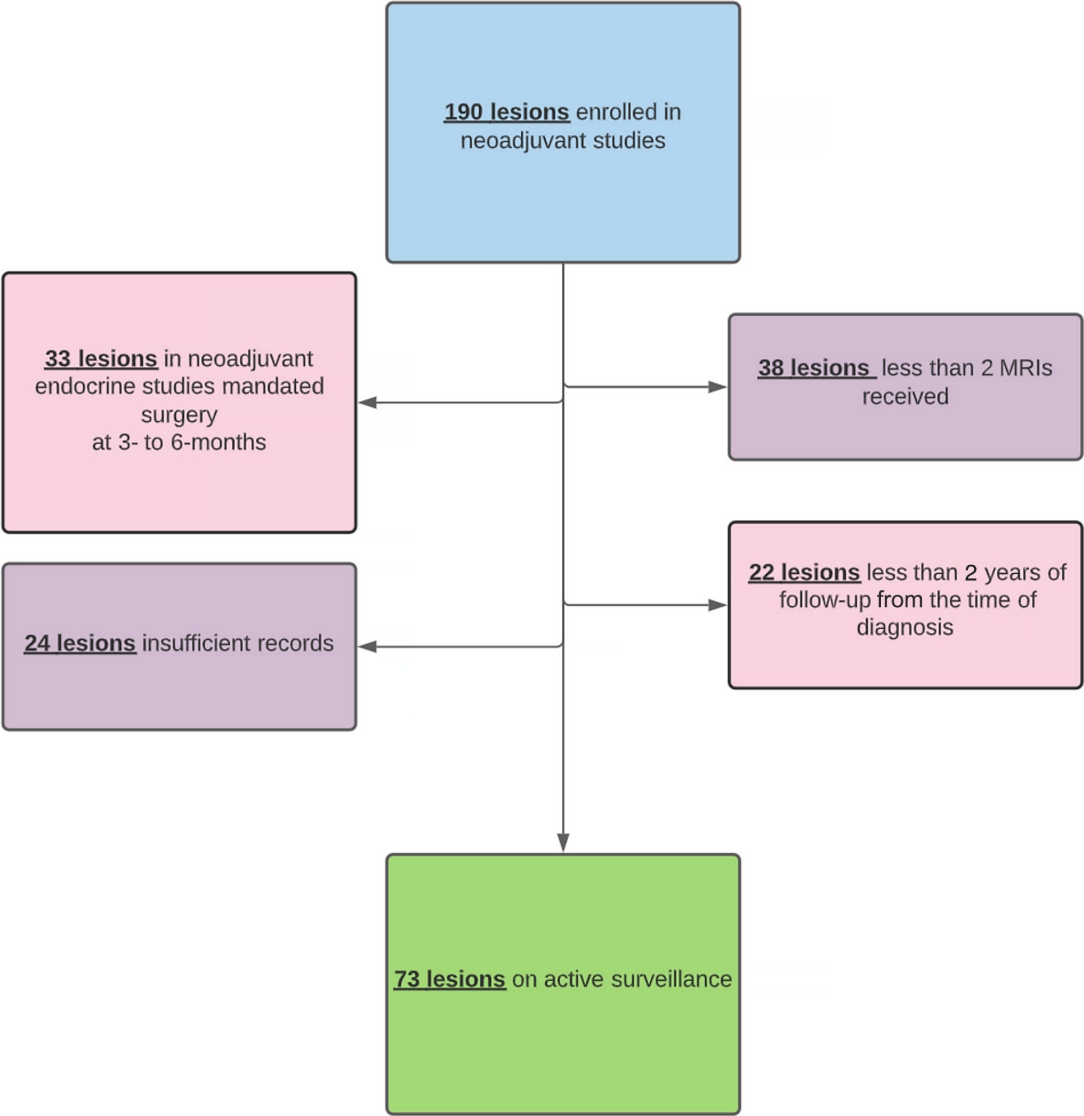
Patients participating in imaging studies who were included in the active surveillance cohort.

The database contained 188 women (190 lesions, as 2 women had bilateral DCIS) diagnosed between 2002 and 2019 who received an MRI under one of the imaging protocols. In a retrospective review of this cohort, we excluded 33 women (33 lesions) as they were on imaging protocols in which surgery was mandated, 38 women (38 lesions) who received fewer than two MRIs, 24 women (24 lesions) who had insufficient records, and 22 women (22 lesions) who had less than 2 years of follow-up from the time of DCIS diagnosis. In total, 117 lesions were excluded. In our final cohort, 73 lesions (71 women, 2 had bilateral DCIS) met the criteria for inclusion in the retrospective analysis (Fig. 1).

Table 1 shows demographic and clinical characteristics of our cohort. The average age at the time of diagnosis was 53.8 years (range, 29.8–78.8 years); 46.6% of the DCIS lesions (34/73) occurred in women who were premenopausal. 93.2% (68/73) of the lesions were HR^+^ with endocrine therapy offered to all of these women (89% accepted). Mean follow-up time was 8.5 years (range, 2.1–21.5 years).

**TABLE 1:** Demographics and clinical characteristics of the active surveillance cohort at the time of initial presentation with DCIS

Characteristic	Value (*n* = 73)
**Mean age at diagnosis** (range), years	53.8 (29.8–78.8)
29.0–39.0	1 (1.4%)
40.0–49.0	26 (35.6%)
50.0–59.0	31 (42.4%)
60.0–69.0	9 (12.3%)
70.0–79.0	6 (8.2%)
**Race**	
Asian	10 (13.8%)
Black	2 (2.7%)
White (Non-Hispanic)	47 (64.4%)
Hispanic	4 (5.5%)
Other	5 (6.8%)
Decline	5 (6.8%)
**Mean follow-up** (range), years	8.5 (2.1–21.5)
**Mean time on AS total** (range), years	4.78 (0.2–19.3)
**Mean time on AS before surgery** (range), years	2.1 (0.2–5.8)
**Mean time on AS and no surgery** (range), years	7.4 (2.1–19.3)
**Menopausal status**	
Premenopausal	34 (46.6%)
Postmenopausal	39 (53.4%)
**HR status**	
Positive	68 (93.2%)
Negative	2 (2.7%)
Unknown	3 (4.1%)
**ER status**	
Positive	68 (93.2%)
Negative	2 (2.7%)
Unknown	3 (4.1%)
**PR status**	
Positive	60 (82.2%)
Negative	7 (9.6%)
Unknown	6 (8.2%)
**HER2 status**	
Positive	9 (12.3%)
Negative	39 (53.4%)
Unknown	25 (34.2%)
**Grade**	
High	23 (31.5%)
Intermediate	37 (50.6%)
Low	11 (15.1%)
Unknown	2 (2.7%)
**Endocrine therapy offered**	
Yes	71 (97.3%)
Not documented	2 (2.7%)
**Endocrine therapy accepted**	
Yes	63 (89.1%)
No	10 (10.9%)
**Surgery**	
Yes	33 (45.2%)
No	40 (54.8%)

**FIGURE 2:**
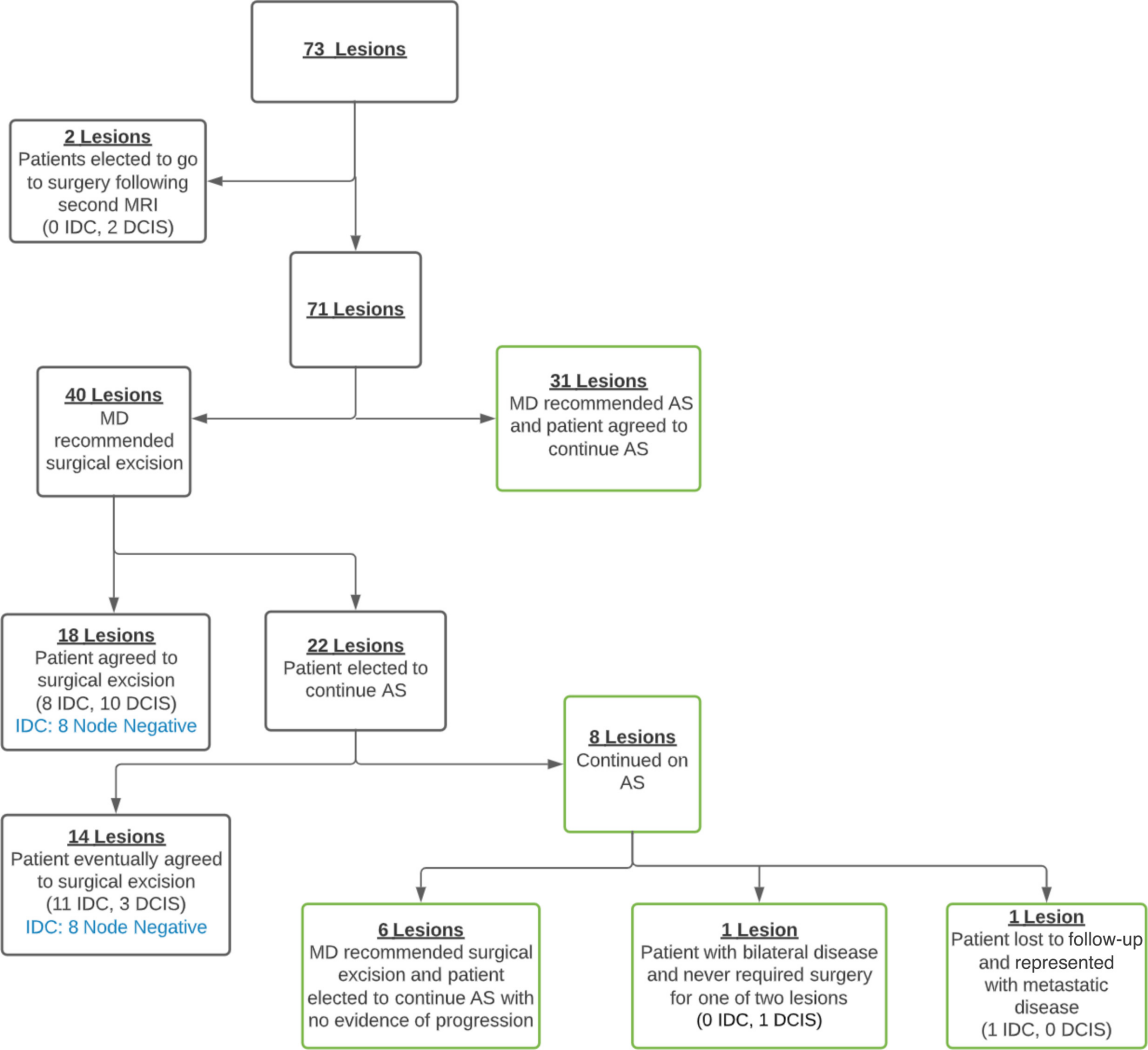
Active surveillance treatment diagram.

A total of 52.1% (37/71) of women in our cohort representing 38 lesions remain on active surveillance (AS) with a mean duration of 7.4 years (range, 2.1–19.3 years). A total of 46.6% (34/73) of lesions were treated with surgical resection more than 6 months after initial diagnosis. Progression of disease was suspected for 22 lesions with surgery recommended but not immediately followed per patient preference. The majority of this group (14/22 lesions), but not all, ultimately underwent surgical resection (Fig. 2).

**FIGURE 3:**
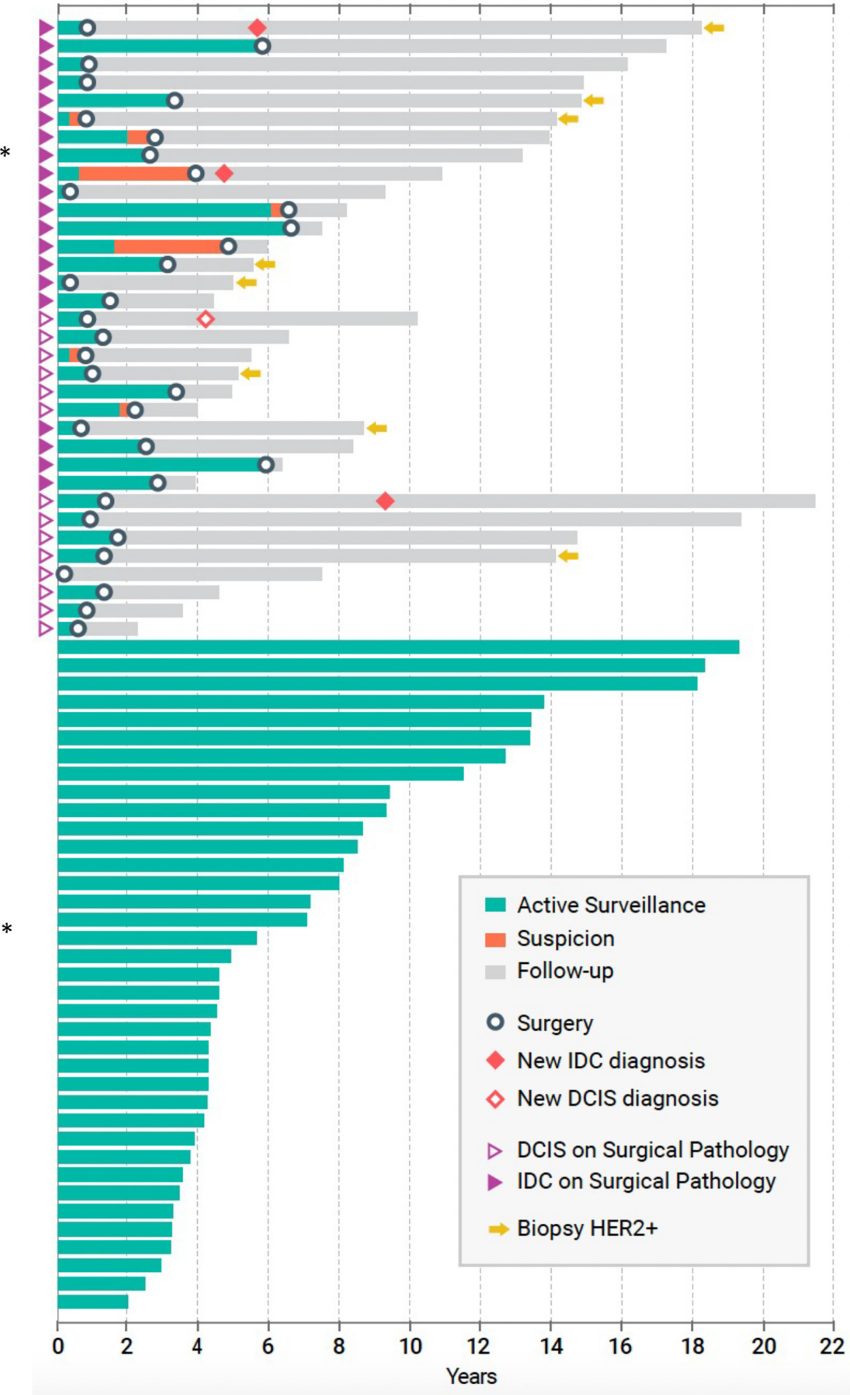
Individual temporal outcomes of each patient in the active surveillance cohort.

Figure 3 illustrates the time course and outcomes of the 71 active surveillance patients with 73 lesions. Of the 34 lesions (34 patients) that underwent surgical excision, 19/34 (55.6%) had IDC, and 15/34 (44.1%) had DCIS. Of the 20 lesions (20 patients) with IDC on surgical pathology (including 1 patient lost to follow-up who later presented with metastatic disease), the surgeon noted concern for progression and recommended excision. The time course for noting progression varied, but the majority of lesions (80%) showed signs of progression clinically or by imaging within 4 years with 55% of lesions within 2 years, 25% of lesions between 2 and 4 years, and 15% of lesions between 4 and 6 years from the start of active surveillance. The incidence of IDC was not higher for lesions identified in premenopausal (23.5%, 8/34) than in postmenopausal (30.8%, 12/39) women. There were 3 women who developed new ipsilateral IDC after surgical resection and 1 with new DCIS, all of whom underwent surgery for their recurrences. A total of 37 women (38/73 lesions, 52.1%) remain on active surveillance without evidence of invasive disease.

**FIGURE 4:**
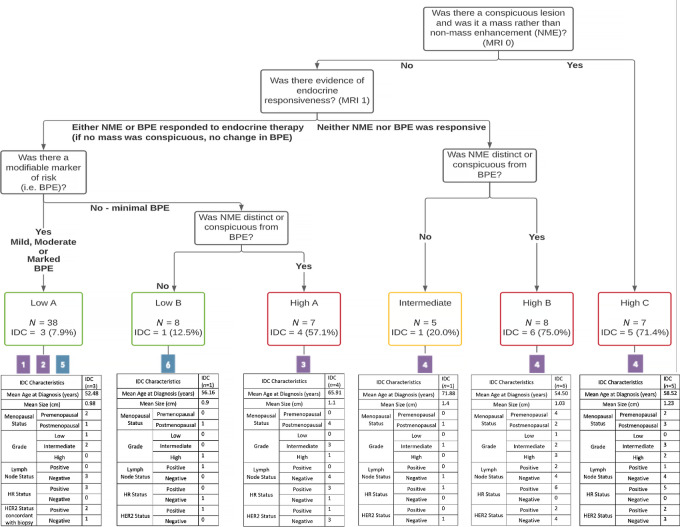
Retrospective analysis using a recursive partitioning (R-PART) classification of DCIS lesions.

**FIGURE 5:**
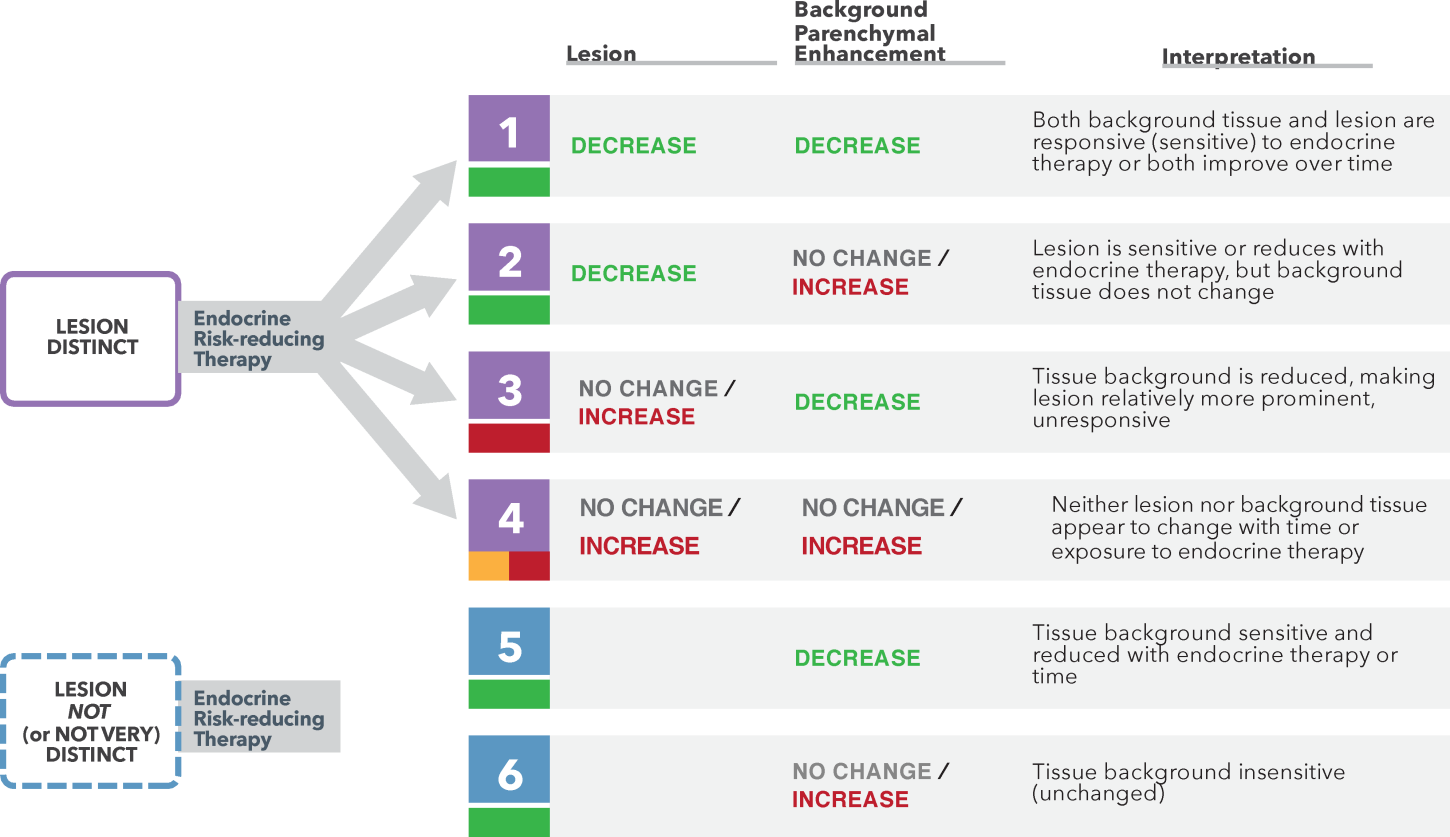
Implications of lesion and BPE changes on MRI over time.

R-PART was a tool used to retrospectively identify which imaging features were most predictive of developing IDC in the setting of active surveillance. The following features, identified independently by two separate radiologists, were found to be most prognostic: lesion distinctness, whether the lesion was a mass versus NME, and categorization of BPE as minimal, mild, moderate, or marked (Fig. 4). Six separate risk categories (low A/B, intermediate, high A/B/C) were developed on the basis of results from the retrospective partitioning analysis and the features prioritized by this algorithm. Of the 46 lesions classified as low-risk (low A and low B), four (8.7%) progressed to IDC; one of these IDC lesions was HR negative (HR^−^), and three were HER2 positive. The patient in the low-risk group who developed IDC with positive nodes had not followed the advice of her surgeon to undergo resection at the time of an imaging change. In the intermediate-risk group, one lesion progressed to node-negative, HR^+^, HER2-negative IDC. In the high-risk category, 15 of 22 lesions (68.2%) had IDC on surgical pathology; one of these IDC lesions was HR^−^, and five were HER2 positive. Three of the IDC lesions that developed in the high-risk group were node positive with all of them occurring in patients who continued active surveillance against surgeon advice. Of the 7 patients with HR^+^ DCIS who did not take endocrine therapy (ET), 3 developed IDC. The risk partitioning algorithm was also run with grade and lesion size as input variables, and these did not impact the partitioning of the patients. Our hypotheses for what we observed on MRI are shown in Fig. 5, and the groups in Fig. 4 are numbered on the basis of our conclusions in Fig. 5 to correlate these interpretations with the R-PART findings.

Of the 20 IDC lesions (20 patients), six were HER2 positive and all in postmenopausal patients. All 20 women with IDC are alive. In 14 of 20, initial core biopsy tissue was available and stained for HER2; HER2 concordance was 100% (Table 2). We were not able to obtain all diagnostic core biopsy specimens; however, we estimate in retrospect that 23.2% of the cohort was HER2 positive based upon 56 specimens with available HER2 results (Supplementary Tables S1 and S2). Molecular profiles were successfully generated on 12 IDC lesions (12 patients); five were luminal A, five luminal B, and two HER2 type (Table 2). The only lesion that was ultralow risk based on molecular profile was also HR^+^ HER2-positive and luminal A.

**TABLE 2:** HER2 status for patients who had IDC at the time of surgical excision

	DCIS on biopsy				IDC at surgery						
Block	ER (%)	PR (%)	HER2 IHC/FISH	Location: Laterality/Quadrant	ER (%)	PR (%)	HER2 IHC/FISH	Location: Laterality/Quadrant	Agendia MammaPrint risk	BluePrint type	MammaPrint index
HER2 status concordance on core biopsy and surgical resection specimen											
HER2-positive status concordant on core biopsy and/or surgical resection specimen											
UCSF_175	100	90	3+	Left/UOQ	99	15	3+	Left/UOQ	Low	Luminal A	0.402
UCSF_098	99	2	3+	Right/LOQ	95	0	3+	Right/LOQ	High	Her2	−0.189
UCSF_078	0	0	3+	Left/multifocal	80	1	3+	Left	High	NA	NA
UCSF_149	0	0	3+	Left/UIQ	0	0	3+	Left	NA	NA	NA
UCSF_009	90	90	3+	Left/central	20	0	3+	Left/central	NA	NA	NA
UCSF_030	0	0	NA	Left/LIQ	40	0	3+	Left/LIQ	High	Her2	−0.3
HER2-negative status concordance on core biopsy and surgical resection specimen											
UCSF_087	95	10	1+	Left/UIQ	100	0	2+	Left/UIQ	QNS	QNS	QNS
UCSF_100	95	30	1+	Right/UIQ	90	0	0	Right/UIQ	Low	Luminal A	0.172
UCSF_031	90	80	0	Right/UOQ	50	0	1+	Right/UOQ	NA	NA	NA
UCSF_076	90	60	0	Left/UOQ	0	0	1+	Left/Upper Central	Low	Luminal A	0.157
UCSF_096	95	95	0	Right/UOQ	90	95	1+	Right/UOQ	High	Luminal B	−0.429
UCSF_116	95	95	0	Right/UOQ	95	95	0	Right/UOQ	NA	NA	NA
UCSF_162	95	10	0	Right/UOQ	100	0	0	Left/UOQ	High	Luminal B	−0.264
UCSF_106	100	20	2+	Left/UOQ	>90	0	2+	Left	High	Luminal B	−0.217
UCSF_012	95	95	NA	Left/multifocal	95	20	2+	Left/Multifocal	NA	NA	NA
UCSF_042	95	95	NA	Right/UIQ	100	100	1+	Right/UOQ	Low	Luminal A	0.336
UCSF_197	80	90	NA	Left/12 o'clock	90	50	2+	Left/UOQ	Low	Luminal A	0.063
UCSF_191	0	0	NA	Left/UIQ	95	0	2+	Left/UOQ	QNS	QNS	QNS
UCSF_052B	0	0	No Tissue	Right/UIQ	95	0	2+	Right/UIQ	High	Luminal B	−0.094
HER2-negative status for metastatic disease and no surgical resection											
UCSF_072	90	90	1+	Left/UOQ	Bilateral >90%	right - 1%	2+	Bilateral	High	Luminal B	−0.525

## Discussion

This registry study provided us with an opportunity to retrospectively evaluate outcomes of a unique cohort of patients who declined surgical resection of their DCIS and, instead, elected to undergo active surveillance with endocrine therapy and intensive serial breast MRI. This analysis suggests that changes in MRI features after a 3- to 6-month trial period of endocrine therapy can be used to stratify the risk of developing IDC. Two key features were identified by clinicians over time and confirmed by the risk partitioning algorithm. The first was the presence of a focal lesion versus generalized enhancement. The second was the presence of a focal mass versus NME. Consistent with findings from BI-RADS, a focal lesion was more likely to be associated with an invasive cancer (21). We hypothesize that the risk partitioning algorithm can be explained by endocrine responsiveness or lack thereof in both the background enhancement as well as the lesion. Given that these features are easily assessed with MRI, we are developing a prospective national study to validate our model for risk assignment and management.

Overdiagnosis and overtreatment of screen-detected cancers is an area of concern with DCIS being a prominent example (1, 22). Current risk assessment tools have not been sufficient to drive specific treatment choices. In 2012, an expert group recommended to the NCI that a strategy of observation over time could be used to determine specific risk for lesions of uncertain invasive potential (4). The R-PART analysis that we developed based upon retrospective analysis of imaging features in response to endocrine risk–reducing therapy in this study cohort demonstrates the potential power of a neoadjuvant and observational approach to guide DCIS therapy.

In this cohort of 71 patients (73 lesions) with variable pathology and imaging findings, the majority (52.1%) of patients were successfully able to undergo active surveillance without development of IDC. We found that different patterns emerged defined by lack or presence of a focal lesion on MRI with variable concordance to mammographic findings. We also identified variable responses of lesions and BPE to endocrine therapy. R-PART was retrospectively applied to our cohort to select MRI-based features that apportion risk for developing IDC. Importantly, the algorithm was designed to refine risk using an early endpoint, 6 months of endocrine therapy exposure. At this early endpoint, we were able to distinguish a high-risk group of women with a 68.2% risk of future IDC from a low-risk group with an 8.7% risk of future IDC with endocrine therapy alone.

Of the lesions that did not show a distinct mass, the MRI features can be interpreted as endocrine responsive versus nonresponsive states, characterized by the development, persistence, or reduction in NME relative to BPE, which we describe in Fig. 5. A key distinguishing feature of the lowest-risk group was the absence of a distinct lesion on MRI. For these lesions, the risk of recurrence or development of IDC conferred by DCIS may be global (anywhere in the breast) rather than focal, more akin to atypia. Interestingly, there is variability among pathologists in differentiating atypia from low- and intermediate-grade DCIS (23, 24). The NSABP B-24 trial established that the addition of adjuvant tamoxifen following lumpectomy and radiation in patients with DCIS reduces the risk of developing ipsilateral invasive breast cancer by 32% and the risk of developing contralateral invasive breast cancer by 53% (9). More recently, DeCensi and colleagues tested low-dose tamoxifen (5 mg/day) in a randomized controlled trial (RCT) enrolling patients with DCIS, lobular carcinoma in situ (LCIS), or atypical ductal hyperplasia (ADH) and found that the ipsilateral DCIS or invasive recurrence rate was decreased by 52% and contralateral recurrence rate decreased by 75% (25). DCIS without a focal lesion on MRI may represent an indicator of bilateral risk and an opportunity to use endocrine therapy rather than surgical resection to decrease that risk.

Our MRI-based R-PART analysis suggests that a conspicuous mass lesion is associated with a relatively high risk of having or developing IDC. This has been observed previously and is a distinguishing feature in the BI-RADS classification system (16, 21). The next most important feature is the pattern of endocrine responsiveness. NME in the setting where neither the lesion nor the BPE responds to ET indicates high risk, unless the NME is not distinct relative to the background. If the BPE is reduced and NME becomes more conspicuous, the risk is elevated, which may indicate that the primary lesion has become independent from endocrine control. NME in the setting of minimal BPE (no modifiable background risk) is also an indicator of greater risk for developing IDC. In contrast, evidence of endocrine responsiveness measured by a decrease in either the lesion/NME or BPE, if present, signals low risk for IDC development. The only setting in which failure to respond to endocrine therapy was not an indicator of risk was when a lesion was not present and BPE was minimal. This clearly demonstrates that there is an interaction between the lesions and the background in which they arise. Our data suggest that we can use short-term exposure of 3 to 6 months of endocrine therapy and serial MRI to categorize or modify the risk for developing IDC and a patient's suitability for long-term active surveillance.

There was a relatively large number of patients with high-risk features in our active surveillance cohort. Indeed, 37.0% (27/73) of lesions were in patients under the age of 50 and 46.6% (34/73) were in premenopausal patients; 31.5% (23/73) of lesions were high grade, and (in retrospect) 23.2% (13/56) were HER2 positive. Given our cohort consisted of a group of patients with diverse biology united by their desire to avoid surgery unless there was clear evidence of invasive breast cancer, this cohort differs from those currently enrolled on three phase III RCTs evaluating active surveillance compared with standard treatment of DCIS; the patients enrolled in these RCTs must be ≥45 years old and have low-grade, screen-detected nonpalpable DCIS without a discrete mass (26–29). Yet despite the higher risk nature of our cohort, over 50% of patients have been able to avoid surgery without development of IDC with a long duration (mean 8.5 years) of follow-up. Importantly, our low-risk group identified by R-PART included more patients with high-grade DCIS (30.4%) and intermediate-grade DCIS (43.5%) than low-grade DCIS (21.7%). As more data emerge from the ongoing low-risk RCTs, more options are likely to arise for women with lower risk DCIS lesions.

While we were missing some data, concordance between the available HER2 status of core biopsies and surgical resection specimens (either IDC or DCIS) was 100% (14/14). A total of 30% of IDC lesions that developed were HER2 positive. A total of 23.2% (13/56) of lesions with available tissue were HER2 positive at diagnosis, and about half did not progress to invasive cancer. Currently, HER2 testing is not routinely performed on DCIS for use in clinical decision-making. However, in the setting of active surveillance, we find that this marker might improve our ability to elucidate the role of HER2 in subsequent IDC development. Our data are insufficient to determine whether HER2-positive status should be an exclusion criterion for active surveillance. Interestingly, trastuzumab alone has not been shown to be an effective strategy for DCIS (30), and it is, therefore, important to better define the biology to develop more targeted strategies for this subtype.

Molecular subtyping of the IDC that developed revealed luminal A, B, and HER2 type. We did not identify basal types. Interestingly, in the setting of endocrine therapy, there were more luminal B cases that developed. Without full subtyping of the DCIS, it is not possible to know whether the higher rates of luminal B invasive cancers reflect the emergence of endocrine resistance (therapeutic pressure) or whether the luminal B tumors are the ones least likely to respond to endocrine therapy (intrinsic biology at diagnosis). The concordance of HER2 in this series are in keeping with other studies (31, 32) and suggest that there are intrinsic differences between DCIS lesions that can and should be identified at diagnosis and that may help us to improve management in the future. It is likely that DCIS subtyping could forecast the invasive cancer that develops, and molecular characterization may inform improved treatment, including those at risk for luminal B tumors. This highlights the importance of translational studies in the DCIS setting. We have developed a protocol for investigating this and other hypotheses about endocrine resistance and sensitivity using patient-derived organoid cultures and hope these studies will be able to inform management in the future.

Overall, when comparing the rate of recurrence in our cohort to the national rates established by the NSABP B-24 trial following lumpectomy alone or lumpectomy and radiation for DCIS, our low-risk group experienced an ipsilateral IDC rate (8.7%) with endocrine therapy alone that was lower than the ipsilateral IDC rate following lumpectomy alone (19.4%) and similar to that following lumpectomy and radiation (8.9%; ref. 9). Our data include 7 patients with HR^+^ disease who elected not to receive endocrine therapy with 3 of them found to have IDC at the time of surgical resection.

Serial MRI provides a means to evaluate the efficacy of endocrine therapy in preventing progression of DCIS. A short-term metric or early endpoint to assess efficacy of an intervention is one of the most critical elements that can drive early adoption to prevent long-term consequences. For early-stage high-risk invasive breast cancer, we have demonstrated that pathologic complete response is an excellent predictor of recurrence-free survival. Imaging and pathology in the neoadjuvant setting can be used to optimize response to prevent metastatic disease (33). In DCIS, we propose that MRI features be used as early endpoints to predict the risk of future IDC and to optimize management. The baseline features including the identification of a focal mass and extent of BPE can be combined with the relative reduction of BPE and NME following short-term exposure to endocrine therapy. These have the potential to predict the development of IDC in the future. Importantly, these imaging tools may provide a platform for tailoring treatment to biology. DCIS should be considered an important opportunity to pilot strategies for risk reduction, cancer interception, and prevention.

This study is limited by its nonrandomized design and need to develop a pragmatic approach. However, willingness to follow patients who were unwilling to pursue a surgical approach provided an opportunity to improve our understanding of the natural history of DCIS. Similar studies have led to practice change in other settings, such as prostate cancer and Barrett's esophagus (34, 35). Another limitation of our study is related to those patients who elected to delay surgical intervention for several months to years despite clinical suspicion for progression; in these patients, the timing of progression could only be ascertained at surgical resection, though it likely occurred earlier. While there were 7 women with HR^+^ DCIS who elected never to start endocrine therapy, it is also possible that there were women who began endocrine therapy but subsequently discontinued it due to intolerable adverse effects; it is a limitation of this study that we were not able to determine how many women experienced toxicity of endocrine therapy that led to early discontinuation. Finally, we were not successful in obtaining all tissue blocks retrospectively, and this limited our ability to fully characterize the entire cohort. This emphasizes the need for prospective tissue collection in future studies.

Overall, this study suggests that a significant proportion of women diagnosed with DCIS may be able to safely manage their disease with endocrine therapy alone without surgery; those identified as low-risk using our risk partitioning algorithm have a risk of IDC lower than that following lumpectomy alone and similar to that following lumpectomy and radiation. Prospective randomized trials of surgery versus endocrine therapy for lower risk DCIS are ongoing (29) and will demonstrate the rates of IDC development on each arm as well as time to surgery. Our retrospective study identified MRI features that may be able to be used to optimally select those more suitable for active surveillance. MRI in the setting of endocrine therapy may distinguish focal from global risk and improve identification of patients with DCIS who benefit most from surgical resection. This study provides a framework for achieving cancer interception and prevention as well as a foundation upon which to build serial improvements to DCIS active surveillance. A prospective validation trial has been developed with enrollment expected to begin in the spring of 2023.

## Supplementary Material

Table S1 Supplementary Table 1

Table S2 Supplementary Table 2
