# Supplementary material for: Identifying Good Candidates for Active Surveillance of Ductal Carcinoma In Situ: Insights from a Large Neoadjuvant Endocrine Therapy Cohort
Source: Cancer Res Commun. 2022 Dec 7;2(12):1579–89. doi: 10.1158/2767-9764.CRC-22-0263 (PMC10035518; doi:10.1158/2767-9764.CRC-22-0263)
Supplement: Table S1 — Supplementary Table 1 [file crc-22-0263-s01.pdf]

Table S1: HER2 Status for Patients who had DCIS at the Time of Surgical Excision

Block ID	DCIS Biopsy				Surgery (DCIS)			
	**ER + (%)**	**PR + (%)**	**HER2 Status IHC/FISH**	**Location** Laterality/Quadrant	**ER + (%)**	**PR + (%)**	**HER2 Status IHC/FISH**	**Location** Laterality/Quadrant
**HER2 Status Concordance on Core Biopsy or Surgical Resection Specimen**								
HER2 Positive Status Concordant on Core Biopsy and/or Surgical Resection Specimen								
UCSF_002	30	50	No tissue	Right/UOQ	95	0	3+	Right/UOQ
UCSF_172	0	0	No tissue	Right/UOQ	95	0	3+	NA
UCSF_095	90	70	NA	Left/multifocal	30	0	3+	NA
UCSF_159	90	50	NA	Left/UOQ	90	90	3+	NA
UCSF_041	2	1	3+	Right/UIQ			NA	Right/UIQ
UCSF_178	90	0	3+	Left/Multifocal			NA	NA
HER2 Negative Status Concordant on Core Biopsy and Surgical Resection Specimen								
UCSF_190	90	90	2+	Left/central	99	99	0	NA
UCSF_081	95	85	1+	Right/UOQ	90	50	1+	Right/UOQ
UCSF_192	99	99	NA	Left/UOQ	90	90	1+	Left/UOQ
UCSF_033	98	98	NA	Left			0	Left/LOQ
UCSF_133	0	0	No tissue	Left/LOQ	80	60	2+	Left/LOQ
UCSF_104	100	2	NA	Right/central			2+	bilateral
UCSF_171	100	0	NA	Left/UIQ			NA	Left
UCSF_107	95	95	NA	Left/UOQ			NA	Left
UCSF_083	95	0	1+	Left/UOQ	NA	NA	NA	Left/UOQ
