# Supplementary material for: Identifying Good Candidates for Active Surveillance of Ductal Carcinoma In Situ: Insights from a Large Neoadjuvant Endocrine Therapy Cohort
Source: Cancer Res Commun. 2022 Dec 7;2(12):1579–89. doi: 10.1158/2767-9764.CRC-22-0263 (PMC10035518; doi:10.1158/2767-9764.CRC-22-0263)
Supplement: Table S2 — Supplementary Table 2 [file crc-22-0263-s02.pdf]

Table S2: HER2 Status for Patients who Continued Active Surveillance without Surgery

Block ID	DCIS Biopsy			
	**ER + (status/%)**	**PR + (status/%)**	**HER2 Status IHC/FISH**	**Location Laterality/Quadrant**
UCSF_164	85	15	3+	Right/UOQ
UCSF_043	60	90	2+	Left/LIQ
UCSF_080	90	90	2+	Left/LOQ
UCSF_099	90	15	2+	Left/central
UCSF_158	0	0	2+	Left/UOQ
UCSF_187	95	25	2+	Left/LIQ
UCSF_101	95	80	2+	Left/LIQ
UCSF_182	positive	positive	2+	Right/LOQ
UCSF_166	100	20	0	Left/UIQ
UCSF_195A	90	20	0	Left/UOQ
UCSF_077	95	30	1+	Left/UOQ
UCSF_103	positive	positive	1+	Right/multifocal
UCSF_161	95	95	1+	Right/LOQ
UCSF_168	100	10	1+	Right/multifocal
UCSF_169	0	0	1+	Right/UOQ
UCSF_173	100	0	1+	Right/central
UCSF_183	positive	positive	1+	Left/multifocal
UCSF_184	positive	positive	1+	Right/LOQ
UCSF_185	90	80	1+	Right/UIQ
UCSF_195B	99	98	1+	Right/LOQ
UCSF_008	85	40	1+	Left/UIQ
UCSF_055	95	60	1+	Right/UOQ
UCSF_200	90	90	0	Right/LIQ
UCS_052A	95	0	No tissue	Left/multifocal
UCSF_086	positive	positive	No tissue	Right/UIQ
UCSF_160	positive	positive	No tissue	Right/LOQ
UCSF_167	positive	positive	No tissue	Left/LOQ
UCSF_060	NA	NA	NA	Right/multifocal
UCSF_097	90	80	NA	Right/UOQ
UCSF_003	30	50	NA	Rght/UOQ
UCSF_044	positive	positive	NA	Right/UIQ
UCSF_047	90	20	NA	Left/LOQ
UCSF_163	positive	positive	NA	Left/UIQ
UCSF_186	positive	positive	NA	Left
UCSF_193	90	30	NA	Right/multifocal
UCSF_198	positive	positive	NA	Right/LQ
UCSF_199	95	95	NA	Right/UOQ
UCSF_201	95	80	NA	Right/UOQ
